# Immunophenotypic Panel for Comprehensive Characterization of Aggressive Thyroid Carcinomas

**DOI:** 10.3390/cells14191554

**Published:** 2025-10-06

**Authors:** Mihail Ceausu, Mihai Alin Publik, Dana Terzea, Carmen Adina Cristea, Dumitru Ioachim, Dana Manda, Sorina Schipor

**Affiliations:** 1 Department of Pathology, “C.I. Parhon” National Institute of Endocrinology, 011863 Bucharest, Romania; mihail.ceausu@umfcd.ro (M.C.); danaterzea@gmail.com (D.T.); carmen.adina83@yahoo.com (C.A.C.); dumitru_ioachim@yahoo.co.uk (D.I.); 2 Department of Pathology, “Carol Davila” University of Medicine and Pharmacy, 022328 Bucharest, Romania; 3 Department of Research, “C.I. Parhon” National Institute of Endocrinology, 011863 Bucharest, Romania; dana.manda@parhon.ro (D.M.); sorina.schipor@parhon.ro (S.S.)

**Keywords:** aggressive thyroid carcinoma, poorly differentiated thyroid carcinoma, anaplastic thyroid carcinoma, BRAFV600E, TTF-1, TERT, PAX-8, K-RAS, TP53

## Abstract

Aggressive thyroid carcinomas—anaplastic (ATC) and poorly differentiated (PDTC)—are rare but highly lethal malignant entities. Their immunophenotypical characterization is still incomplete, and no standardized diagnostic algorithms have been used. Our study retrospectively analyzes 40 thyroidectomy cases as follows: 12 ATC and 28 PDTC from 2014 to 2024 by evaluating clinical data, histopathological aspects, molecular analysis for presence of BRAFV600E and TERTC228/250T mutations, as well as immunohistochemical expression of BRAF_V600E_, total BRAF, K-RAS, TERT, PAX-8, TTF-1, P53, and Ki-67. BRAF_V600E_ was present in 70% of cases, with higher prevalence in ATC. Total BRAF correlated positively with K-RAS and TERT and negatively with BRAF_V600E_. TERT abnormal expression was highly prevalent in over 90% of cases, while loss of TTF-1 and PAX-8 is associated with anaplastic transformation. Ki-67 proliferative index had significantly higher values in ATC, thus supporting its role as a marker for aggressiveness. On univariate analysis, higher Ki-67 indices and lymph node invasion are independent predictor factors for the presence of metastases. However, on multivariate analysis, they both lose significance. Upon multivariate analysis, loss of TTF-1 and tumor necrosis were significant predictors for anaplastic histotype. Specific BRAF_V600E_ immunohistochemistry may be a good screening tool for the BRAF_V600E_ mutation. Molecularly, there is a relatively frequent association of the BRAFV600E mutation and TERTC228, mainly in the PDTC subgroup. Patterns of marker expression suggest that BRAF or RAF activation with subsequent loss of TTF-1 or PAX-8, TERT upregulation, and TP53 alteration are frequent occurrences in aggressive thyroid carcinomas. The association between TTF-1 loss and anaplastic transformation, presence of necrosis alongside BRAF_V600E_, underlines their diagnostic potential in subclassifying aggressive thyroid carcinomas.

## 1. Introduction

Global Cancer Observatory ranks thyroid cancer as the seventh malignancy in incidence, cumulating a total of 821.214 new cases yearly. It ranks position 24 in mortality, accounting for 47.507 deaths in 2024 [1]. Incidence rates and tumor size have been growing substantially in the last 20 years, while diagnosis age has been decreasing [2]. Pediatric thyroid cancers are rising in incidence and nodule dimension upon diagnosis [3,4]. Higher dimension nodules and higher incidence of late stage at diagnosis suggest etiologic factors as the drivers for this increase, and to a lesser extent, the overdiagnosis [5]. Despite intensive efforts such as public health policies, efficient screening programs, and better treatment, mortality rates remain the same or even slowly increase [6].

As recognized by the 5th WHO Classification of Tumors, by far the rarest subtypes of thyroid malignancy are represented by high-grade follicular cell-derived non-anaplastic thyroid carcinoma and anaplastic thyroid carcinoma (ATC), with cumulative frequencies of 2–10% of all thyroid malignancies [7,8,9]. The first category encompasses the newly introduced differentiated high-grade thyroid carcinoma and poorly differentiated thyroid carcinoma (PDTC) [10]. PDTC and ATC are aggressive types due to the poor prognosis, low clinical outcome, and restricted treatment options [11]. In their majority, ATC and PDTC show radioiodine refractoriness due to the loss of sodium-iodine symporter (NIS) on the cell membrane, thus not importing the I^131^ [12]. For comparison, differentiated thyroid carcinomas have excellent 5-year survival rates of over 95%, whereas PDTC exerts 78% 5-year survival rates and ATC has a grim 2-year survival rate of only 11.5%, generating a significant burden of over half the thyroid-related deaths each year [11,13,14]. Moreover, AJCC (American Joint Committee for Cancer) Staging defines ATC as stage IV, disregarding tumor dimension or age. PDTC, on the other hand, is classified as I or II in patients less than 55 years old, and I to IV for people older than 55, in regard to tumor dimensions, lymph node status, and metastasis. The AJCC system is now challenged by new and revised alternative systems [15].

For the diagnosis of PDTC, Turin criteria is the actual standard, but it includes no immunohistochemical or molecular references [16]. Whereas no immunohistochemical criteria are needed for diagnosis, it is known that mutational events are gathered by the neoplastic cell in a multistep progression [17]. Early driver mutations are considered BRAF and RAS activations (more frequently for PDTC) [5,14,18,19]. Late events are TERT mutations associated with high metastatic potential and TP53 alterations [5,20,21]. As the cell accumulates more genetic aberrations, the more it drifts away from the normal follicular cell, both in behavior and functionality. Additionally, late changes are highly prevalent within ATC patients [20,22].

ATC is considered the endpoint of the neoplastic progression of the follicular cell, gaining both early driver mutations and late driver mutations, completely losing its follicular cell aspect [5]. The majority seems to have a history or histologic indicators of associated differentiated thyroid carcinomas [23,24]. The same multistep progression mechanism is proposed: beginning with BRAF or RAS early mutations and completed by TERT and TP53 late events [14,25,26]. From all these markers, the 5th WHO Classification of Tumors only recommends immunostaining for BRAF_V600E_ due to the anti-BRAF clinically efficient treatments [17,27].

Although the literature comprises many score-reporting systems for different tissue markers, there is a lack of unification when it comes to aggressive thyroid carcinomas. For instance, the literature presents widely variable threshold values for proliferation index regarded as a percentage of Ki-67 tissue expression [28,29,30]. Various immunohistochemistry algorithms for the diagnosis and prognosis of both ATC and PDTC have been proposed, yet the diagnostic criteria in WHO Classification of Endocrine Tumors 5th edition, are scarce in immunohistochemistry [10,31,32,33,34]. The literature mostly explores markers such as PAX-8, TTF-1, P53, TERT, Ki-67, BRAF, and KRAS [14,19,35,36,37,38,39]. Also, specific antibody anti-BRAF_V600E_ mutated protein remains to be validated as a reliable diagnostic marker and as a good predictor for the mutation, as specificity and sensitivity are still variable among studies.

Our study focuses on PDTC and ATC and aims to explore their immunophenotype by means of immunohistochemical analysis, including BRAF_V600E_ as a diagnostic marker, both alone and in comparison with anti-BRAF total protein antibody. We tried to address various marker domains pertaining to different aberrant signaling pathways. Furthermore, we tried to establish a link between various clinical factors and immunohistochemical analysis regarding this aggressive thyroid carcinoma entity seen as a whole.

## 2. Materials and Methods

### 2.1. Case Selection and Study Batch Formation

A total of 40 patients with aggressive thyroid carcinomas were included. Our study retrospectively reviewed “C.I. Parhon” National Institute of Endocrinology archives for patients who underwent total thyroidectomy from 2014 to 2024 and whose pathological diagnosis was PDTC or ATC.

The following selection criteria were used: availability of complete clinical data, paraffin blocks available, histopathologic confirmation of PDTC or ATC, and primary thyroid tumor. Exclusion criteria were tissue from a secondary determination, paraffin block unavailability, fine needle aspiration biopsy, other types of cancer, and lack of or ambiguous clinical data. New or archived tissue slides stained with hematoxylin-eosin were independently evaluated by three of the authors (M.C.C., D.T., and M.A.P.) to confirm the initial diagnosis. For PDTC Turin consensus criteria were applied, whereas for ATC, we respected essential and desirable diagnostic criteria, as per WHO 5th Classification of Tumors [7,33]. The following relevant clinical details were retrieved and included in the study: sex, age, pathologic diagnosis, stage, and lymph node involvement (LNI).

This retrospective study is part of project number PN-IV-P7-7.1-PED-2024-0307, supported by the Ministry of Research, Innovation, and Digitization, CCCDI-UEFISCDI, within PNCDI IV. The project was approved by the Ethics Committee of the Scientific Council of “C.I. Parhon” National Institute of Endocrinology with number 6/04.03.2025. The authors acknowledge and respect the Declaration of Helsinki 1975, as revised in 2013.

### 2.2. Immunohistochemistry

A list of antibody details, dilutions, and manufacturers used for immunohistochemistry is presented in Table 1. For each case, serial 4 μm tissue sections from formalin-fixed embedded tissue were sliced and transferred to adhesive slides. Immunohistochemistry was performed automatically on BOND-MAX Fully Automated IHC and ISH Staining System (Leica Microsystems, Nussloch, Germany, 49.0051), using BOND reagents, according to the producer’s standard protocol. All tissue samples were rapidly fixed in 10% neutral buffered formalin for 24 h.

Briefly, the immunohistochemistry protocol was as follows: dewaxing, heat-induced epitope retrieval with Bond Epitope Retrieval Solution 1 (Leica Microsystems, Deer Park, IL, USA, AR9961) for 20 min at 100 C, 5 min peroxidase block (Leica Microsystems, Deer Park, IL, USA, RE7101-CE), 15 min primary antibody incubation, 8 min post primary, 8 min incubation with Polymer, 10 min incubation with detection system, and 5 min hematoxylin nuclear counterstaining.

Visualization of the tissue-bound antibody was obtained by using BOND Polymer Refine Detection system (Leica Microsystems, Deer Park, IL, USA, DS9800). For BRAF_V600E,_ the protocol was different: heat-induced epitope retrieval with Bond Epitope Retrieval Solution 2 (Leica Microsystems, Deer Park, IL, USA, AR9640) and a longer 30-min primary antibody incubation, as proposed by Szymonek et al. for improved specificity and sensitivity [40].

The immunohistochemical expression of BRAF, K-RAS, TERT, and PAX-8 was scored based on the extent of staining in the tumor cells: a score of +0 means completely negative, +1 for rare cells or isolated positive, +2 focal or zonally positive, and +3 diffusely positive. For TTF-1, P53, and Ki-67, we chose a more detailed semi-quantitative scoring system. TTF-1 was scored as follows: negative for no positive cells; <15%, low; 15–50%, intermediate; >50%, normal. For P53, we used the 10% cut-off value between normal or wild-type (between 0 and 10%) pattern and mutant-type or abnormal pattern (>10%), and also complete absence was considered mutant-type, as described for other cancers [41,42]. For Ki-67, we elaborated a scoring system that tries to unify heterogeneous thresholds used in previous literature for different thyroid entities: <5%, low expression; 5-10%, intermediate; 10-20%, high, and >20% very high [28,29,30]. However, to this date, there are no formal thresholds for the expression of Ki-67. For more details of marker cut-off values and their interpretation, see Table 2.

### 2.3. Molecular Assessment

For mutation analysis, DNA was extracted from formalin-fixed paraffin-embedded (FFPE) tissue sections using blackPREP FFPE DNA Kit (Analytik Jena AG, AJ Innuscreen GmbH, Jena, Germany) according to the manufacturer’s instructions. DNA concentration and purity were assessed using a Denovix spectrophotometer and Qubit 3.0 fluorometer.

PCR amplification of exon 15 of the *BRAF* gene potentially containing the T1799A missense variant (encoding BRAFV600E), was performed using forward primer 5′- GCTTGCTCTGATAGGAAAATGAG -3′ and reverse primer 5′- GTAACTCAGCAGCATCTCAGG -3′. Thermal conditions were optimized and included an initial denaturation step at 95 °C for 5 min, followed by 35 cycles of denaturation at 94 °C for 30 s, annealing at 58 °C for 30 s, extension at 72 °C for 30 s, and a final extension at 70 °C for 7 min.

*TERT* promoter PCR amplification was performed using forward primer 5′- CACCCGTCCTGCCCCTTCACCTT -3′ and reverse primer 5′- GGCTTCCCACGTGCGCAGCAGGA -3′ and the following touchdown thermal conditions: initial denaturation step at 95 °C for 7 min, followed by gradual decrease in temperature (every 1 cycle of denaturation at 95 °C for 15 s, annealing at 74–68 °C for 30 s), and continued amplification by 33 cycles of denaturation at 95 °C, annealing at 67 °C for 30 s, and a final extension at 72 °C for 6 min.

The PCR products were purified using AMPure XP Beads (Beckman Coulter, Inc., USA) and then subjected to bidirectional Sanger sequencing using Dye Terminator Cycle Sequencing (DTCS) Quick Start Kit chemistry on an automated capillary electrophoresis platform (CEQ^TM^ 8000 Genetic Analyzer System, Beckman Coulter, Inc., Fullerton, CA, USA). Chromatograms were analyzed using sequence analysis software (DNA Baser Assembler v5.21.0 (2014), SciVance Technologies, www.DnaBaser.com, accessed on 24 July 2025) and compared to the *BRAF* reference sequence (NM_004333.4) and *TERT* reference sequence (NM_198253.3) obtained from Genome Browser (https://genome.ucsc.edu/, accessed on 24 July 2025). The g.1295228C>T (C228T) and g.1295250C>T (C250T) variants in the *TERT* promoter gene represent cytosine-to-thymine transitions occurring 124 bp (c.-124C>T) and 146 bp (c.-146C>T) upstream of the ATG start codon, respectively, based on the human genome reference GRCh37/hg19.

### 2.4. Statistical Analysis

Statistical analysis was performed using JASP Version 0.19.3, JASP Team (2024). In order to check for normal distribution of variables, we chose the Shapiro–Wilk test, which revealed a non-parametric distribution for the majority. For every reported comparison, statistical significance was tested with either a chi-square test for categorical variables or an independent sample *t*-test for continuous variables. Associations between the cancer type and different clinicopathological parameters were tested using chi-square, and effect size was reported as Cramer’s V. To assess the strength and direction of correlations between different clinicopathological parameters, Spearman’s Rho (ρ) was chosen. Univariate and multivariate logistic regression were employed to evaluate the relationship between the chosen predictors and the outcome variable. The results were reported as odds ratio (OR) and 95% confidence interval (95% CI). For all the statistical analyses, we computed a two-tailed *p*-value, and statistical significance was set at *p* < 0.05.

## 3. Results

### 3.1. Clinicopathologic Assessment

All the available clinicopathological characteristics of the included patients are presented in Table 3. In total, 40 patients were included, 28 being diagnosed with PDTC and 12 with ATC. Five (17.8%) cases of PDTC coexisted with differentiated PTC, whereas no ATC-associated PTC. The majority of patients were female 27 (67.5%), generating a male to female ratio of M:F = 1:2. Mean age within the entire group was 63.7 years, with a range between 39 and 87. Although not statistically significant, the mean age for PDTC and ATC was slightly different: 66.4 and 62.9, respectively.

Considering both tumor subtypes, necrosis was marked in 12 (30%) cases. PDTC demonstrated a low prevalence of necrosis, 5 (17.8%) patients, whereas ATC showed a higher frequency of necrosis in 7 (58.3%) patients (χ2 = 6.55, *p* = 0.010, Cramer’s V = 0.405), suggesting a possible association. Vascular invasion was observed in 35 (87.5%) patients overall, with 25 (89.2%) cases in PDTC and 10 (83.3%) in ATC. Neural invasion, however, was rare, with 12 (30%) patients in the entire cohort, 8 (28.5%) in PDTC, and 4 (33.3%) in ATC. Extrathyroidal disease was present in 30 (81.1%) patients from the entire group, in 22 (78.6%) patients from the PDTC group, and in 8 (88.9%) from the ATC group. Vascular, perineural invasion, and extrathyroidal extension differed slightly, but no statistical significance was achieved. Extrathyroidal disease (pT3B, pT4A, and pT4B) was observed in 29 (78.4%) of the entire cohort and in 19 (73.1%) of PDTC cases and in 10 (90.9%) of ATC cases, where staging was possible. Lymph node involvement was seen in 17 (43.6%) considering both carcinomas, in 12 (48.9%) PDTC patients, and in 5 (41.7%) ATC.

For staging, we used the AJCC Staging System, as recommended by WHO Classification of Endocrine Tumors, 5th edition, and, in parallel, the system proposed by Guo et al. [15] for double referencing. Stage distribution for PDTC patients is 4 (14.3%) in stage I, 13 (46.4%) in stage II, 3 (10.7%) in stage III, and 8 (28.5%) in stage IV. AJCC cancer staging manual classifies all the ATC patients in class IV regardless of tumor dimensions or spread. In our ATC lot, 0 patients were in stage IV-A, 5 in stage IV-B, 2 in stage IV-C, and 5 in unspecified stage IV.

We compared Guo et al. system to the canonical AJCC staging, and we found that they have a coefficient of agreement of 64.3%, best agreement in stage Guo IVA (100%), and the lowest agreement in stage II (46.1%). Most frequently, stage II was classified as III (4 cases) or as Guo IVA (3 cases). Please see Supplemental Figure 1 for the agreement matrix between Guo et al. staging system and the classical AJCC.

### 3.2. Immunohistochemical Analysis

**Figure 1:**
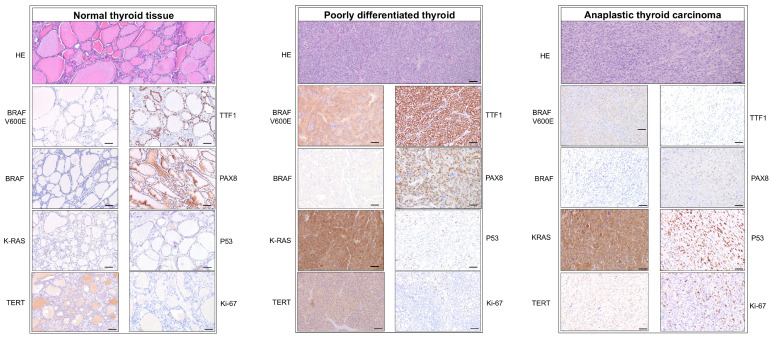
Example of histologic and immunohistochemical expression. (**Left panel**)—expression of markers on normal thyroid tissue: HE (top) shows normal follicular architecture. BRAFV600E is negative; BRAF is negative; TERT is negative in follicular cell nuclei; TTF1 positive; PAX8 positive; P53 wild-type pattern; Ki-67 negative. (**Middle panel**)—photomicrographs of a case of PDTC: HE (top) shows prominent trabecular proliferation with thin fibrovascular septa, cells present minimal pleomorphism and round heterochromatic nuclei without nucleoli. BRAF_V600E_ diffused cytoplasmic staining (+3); B-RAF diffused cytoplasmic staining (+3); K-RAS diffused strong cytoplasmic staining (+3); PAX8 retained nuclear staining; TERT diffused nuclear staining in tumoral cells (3+); TTF1 diffused strong nuclear staining (3+); P53 wild-type pattern: nuclear staining in rare tumoral cells (+0); Ki-67 normal nuclear staining in very rare tumoral cells (+0). (**Right panel**)—photomicrographs of a case of ATC: HE (top) shows spindle cell proliferation (sarcomatoid) with marked nuclear atypia and bizarre hyperchromic nuclei and frequent mitoses; BRAF_V600E_ zonal cytoplasmic staining (+2); B-RAF negative (+0); K-RAS diffused strong cytoplasmic staining (3+); PAX8 rare positive nuclei still retaining expression; TERT zonally positive perinuclear staining (+2); TTF1 negative (+0); P53 mutant-type pattern: positive strong nuclear stain in all tumoral cells (+3); Ki-67 high expression in >10% of tumoral cells (+2). Scale bar = 50 μm.

Immunohistochemical analysis aims to address different markers pertaining to different domains (oncogenic mutational markers, nuclear transcription factors, differentiation markers, proliferation, and aggressiveness) (Figure 1). BRAF and K-RAS are oncogenic mutational markers involved in the MAPK/ERK signaling pathway. BRAF_v600E_ mutant protein was present in 12 (70%) cases from the entire cohort. Six (21%) patients from the PDTC group showed BRAF_v600E_ protein staining, whereas another six (50%) from the ATC group were positive. Chi-square test shows that ATC associates higher BRAF_V600E_ mutant protein activity (χ2=9.388, *p* = 0.025, Cramer’s V = 0.484).

Across all cases, total BRAF elevation was observed in 18 (45%) patients. By tumor subtype, BRAF overexpression was present in 20 (50%) patients pertaining to the PDTC group, and in 4 (33.3%) patients with ATC. However, the difference between the groups was not statistically significant. K-RAS demonstrated an abnormal expression in 16 (60%) cases across all patients. Within the two tumor subtype groups, K-RAS is overexpressed in 12 (43%) of cases with PDTC and in 4 (33%) of ATC. No statistical significance was reached.

**Figure 2:**
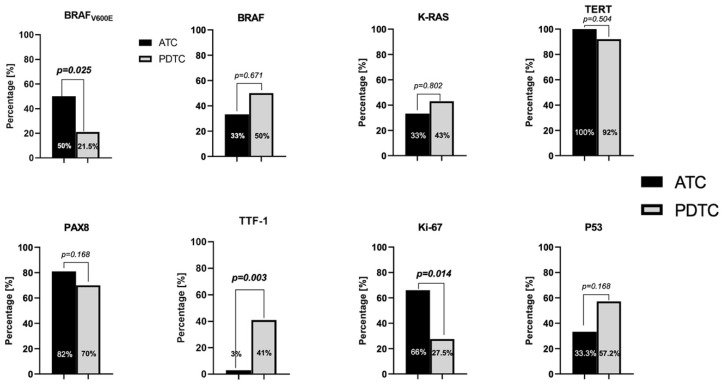
Graphs showing the percentage of abnormal expression of different markers by cancer subtype. ATC—anaplastic thyroid carcinoma; PDTC—poorly differentiated thyroid carcinoma.

TERT and PAX-8 are both nuclear transcription factors linked to the pathogenesis of aggressive thyroid carcinomas. TERT in our lot is overexpressed in 28 cases (95%). By tumor subtype, PDTC cumulates 26 (92%) abnormal expression, whereas ATC demonstrates 12 (100%) patients with increased tissue expression. The difference between the groups did not reach statistical significance. PAX-8 is underexpressed in 30 (75%) cases across both tumor types. PDTC group demonstrates 17 (60%) patients with decreased expression and 3 (10%) patients with no staining. In ATC group 5 (41%), patients had low expression, and another 5 (41%) had absent expression. The difference between the two groups was not significant. Please see Figure 2. for details on differential expression between PDTC and ATC.

TTF1 is another nuclear transcription factor that is used to demonstrate thyroid differentiation in our case, or the alveolar epithelium of the lung. Among all tumor subtypes, 23 (57%) patients displayed normal TTF1 staining. Stratified by diagnostic subtype, PDTC was characterized by 20 (71%) normal levels and 1 (3%) absent staining for TTF1, whereas ATC only displayed 3 (25%) patients with normal expression and 5 (41%) with absent staining. A chi-square test demonstrated a significant association between the tumor subtype and TTF1 expression levels, (χ2=13.88, *p* = 0.003, Cramer’s V = 0.58). Loss or reduction in TTF1 staining was more frequently associated with higher-grade histologic subtype, which may suggest its role as a prognostic and differentiation marker.

Ki-67 is a nuclear protein used as proliferation index in tumor cells. Overall, 19 (47%) patients exhibited higher than normal proliferative indices reflected as increased Ki-67 staining. Within each cancer group, PDTC showed 17 (66.7%) low levels, 11 (27.5%) cases with higher-than-normal proliferative activity, among which 2 (5%) had very high proliferative scores. In contrast, ATC displayed 4 (33%) low index patients, 8 (66%) cases with higher-than-normal Ki-67 scores, among which 5 (41%) had very high index. A strong association was observed between Ki-67 score interpreted as proliferation index and tumor type (χ2 = 10.63, *p* = 0.014, Cramer’s V = 0.516), as tested with the chi-square. In other words, ATC associated higher proliferation indices, consistent with its recognized aggressive histologic behavior.

**Figure 3:**
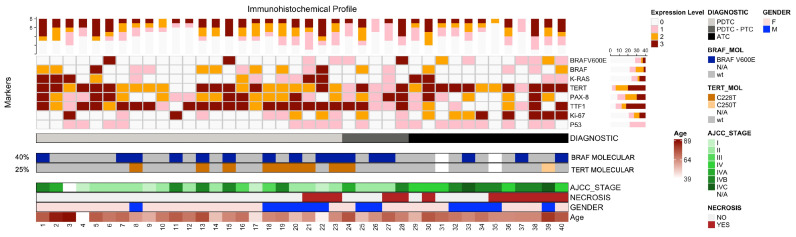
Oncoprint summarizing the clinicopathologic characteristics of the cohort. Each column pertains to a patient from 1 to 40. Each line contains the marker. For each marker, a score between +0 (white) to +3 (dark red) was plotted. Below the oncoprint diagnostic, AJCC stage, presence of necrosis, extrathyroidal extension, gender, and age are plotted as separate lines. ATC—anaplastic thyroid carcinoma; PDTC—poorly differentiated thyroid carcinoma.

P53 is a nuclear tumor suppressor protein involved in the normal cell cycle. In the entire cohort, 20 (50%) patients had a normal wild-type pattern, while 20 (50%) had mutant-type expression (either absent or overexpressed). Considering the histopathologic subgroups, PDTC revealed 12 (42.8%) participants with normal expression and another 16 (57.2%) with abnormal patterns. ATC group, on the other hand, was characterized by 8 (66.6%) cases with wild-type expression pattern and 4 (33.3%) abnormal expression. Despite the noticeable differences between the groups, statistical significance was not reached. A summary of the main clinicopathologic and immunohistochemical characteristics is represented in Figure 3.

### 3.3. Molecular Assessment

Two DNA samples pertaining to two patients have been rejected due to low quality of nucleic acid. Upon analysis, the samples did not meet the minimum quality standards required for reliable testing (degraded DNA). In the entire cohort, 17 (42.1%) of patients harboured the BRAF_V600E_ mutation. In the PDTC subgroup, 12 (42.8%) were positive for the mutation, whereas only 3 (30%) patients were positive in the ATC group. PDTC statistically associates higher BRAF_V600E_ mutation prevalence (χ2 = 7.98, *p* = 0.018, Cramer’s V = 0.447).

Speaking of the TERT mutations, in total, 10 (26.3%) patients presented alterations; specifically, 9 (23.6%) had the C228T mutation, and only 1 (2.6%) patient with ATC had the C250T mutation. When dividing by subgroups, it is clear that the PDTC subgroup significantly associates with higher TERT alterations in 9 (32.1%) patients when compared to ATC, where only 1 (10%) patient presented an alteration (χ2 = 6.63, *p* = 0.036, Cramer’s V = 0.407).

### 3.4. Correlations Between Clinical, Molecular and Pathologic Data

Multiple correlations were observed between oncogenic mutational markers used across all the cases. For a tabular form of the established correlations, please see Table 4. Among our lot components, TTF1 expression and PAX-8 expression showed a robust positive correlation (ρ = 0.711, *p* < 0.001). Total BRAF levels exhibited a moderate positive correlation with K-RAS (ρ = 0.510, *p* < 0.001) and a weak negative correlation with BRAF_V600E_ (ρ = −0.324, *p* < 0.05). Also, a moderate positive correlation was found between total BRAF and TERT (ρ = 0.422, *p* < 0.01). A weak positive correlation between TERT and PAX-8 levels (ρ = 0.323, *p* < 0.05) was found. Ki-67 immunohistochemical expression showed a weak correlation with TERT (ρ = 0.346, *p* < 0.05) and BRAF levels (ρ = 0.350, *p* < 0.05) individually. Multiple linear regression revealed that neither is independently predictive when adjusting for each other.

Statistical correlations between immunohistochemical markers and clinical data were explored. Diagnostic was weakly correlated with Ki-67 expression (ρ = 0.383, *p* < 0.05) and strongly correlated with the presence of necrosis (ρ = 0.405, *p* < 0.01). Ki-67 expression was correlated with the presence of metastases (ρ = 0.432, *p* < 0.05). Also, histopathologic diagnosis showed a strong negative correlation with TTF1 levels (ρ = −0.542, *p* < 0.001), interpreted as a decreased TTF-1 is correlated with anaplastic, more aggressive subtype.

Tissue expression of BRAF_V600E_ mutant protein showed a weak correlation with the age at diagnosis (ρ = 0.325, *p* < 0.05). K-RAS showed a weak correlation with the presence of neural invasive behavior of the tumor (ρ = −0.368, *p* < 0.05). Surprisingly, vascular-invasive tumoral behavior correlates moderately with microscopic extrathyroidal extension (ρ = 0.480, *p < 0.01*). Also, the presence of lymph node involvement in our lot correlated with the presence of metastases (ρ = 0.465, *p* < 0.01).

BRAF_V600E_ mutant protein levels highlighted by immunohistochemistry correlated with the molecular results (ρ = 0.420, *p* < 0.01) and also with the molecular presence of *TERT* C228T mutation (ρ = 0.328, *p* < 0.05). Also, we noticed a strong association between the presence of BRAF_V600E_ mutation and presence of TERT C228T (χ2 = 45.77, *p* < 0.001, Cramer’s V = 0.756). Regarding the PDTC subgroup, seven patients (25%) harbored both mutations simultaneously, whereas non-mutational combination was found in the ATC subgroup.

Logistic regression analysis was carried on to identify potential predictive factors for the histopathologic diagnosis. In the univariate logistic regression models, Ki-67 proliferative index (OR = 2.26, 95% CI [0.19, 1.44], *p* = 0.007), TTF-1 absence (OR = 0.32, 95% CI [−1.85, −0.39], *p* < 0.001), and presence of necrosis (OR = 6.44, 95% CI [0.36, 3.36], *p* = 0.012) were each significant independent predictors for higher-grade diagnosis. However, upon multivariate analysis including all three factors, only TTF-1 (OR = 0.176, 95% CI [−3.09, −0.38], *p* = 0.028) and necrosis (OR = 34.49, 95% CI [0.38, 6.69], *p* = 0.012) remained significant. The association between Ki-67 and diagnosis lost its statistical significance (OR = 2.55, 95% CI [−0.02, 1.90], *p* = 0.057). Same analysis was performed for predicting metastasis, and the only reliable predictor was lymph node involvement (OR = 9.33, 95% CI [0.38, 6.69], *p* = 0.017). For predicting the *BRAF*_V600E_ mutation, specific immunohistochemical staining with anti-*BRAF*_V600E_ antibody proved to be a good predictor (OR = 6.00, 95% CI [1.49, 58.19], *p* = 0.025). We will detail the test characteristics of specific BRAF tissue staining in the next section.

### 3.5. Agreement Between Molecular Techniques and Immunohistochemistry

We aim to assess the performance of the immunohistochemical analysis for BRAF_V600E_ and of TERT, compared to their molecular counterparts. A confusion matrix analysis was performed to compare the immunohistochemical results of the specific anti-BRAF_V600E_ antibody and anti-TERT against the standard molecular Sanger sequencing method.

In the case of BRAF_V600E_, we removed two patients due to the insufficient yield of nucleic acid. In the remaining 38, the immunohistochemical technique showed a sensitivity of 50% and a specificity of 86.4%, indicating that this staining method could efficiently identify the candidate patients for further molecular testing.

**Figure 4:**
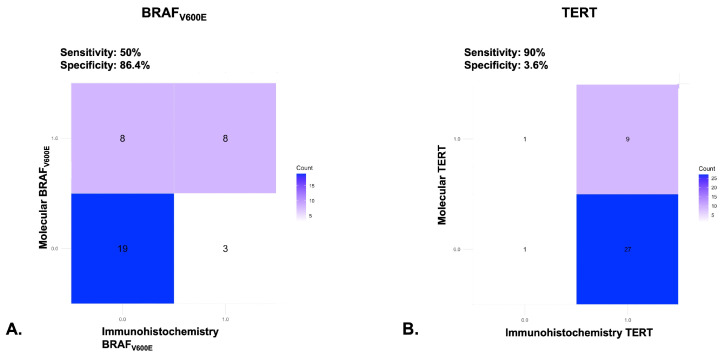
Confusion matrices assessing the diagnostic performance of specific immunohistochemical markers compared to the molecular gold standard method. (**A**) BRAF_V600E_ VE1 clone compared to Sanger sequencing; (**B**) TERT polyclonal compared to Sanger sequencing for C228T and C250T mutations.

Regarding the TERT mutations, after removing the two patients where nucleic acid quality was not adequate, the staining method achieved a sensitivity of 90% and a specificity of 3.6%. This, unfortunately, leads to the conclusion that TERT immunohistochemical staining is not able to adequately identify the corresponding molecular modifications and it cannot be used as a reliable screening method. See Figure 4 for the detailed confusion matrix for both analyses.

## 4. Discussions

Our study explores the immunophenotype of aggressive thyroid carcinomas and aims to elaborate a panel that evaluates the immunohistochemical expression of markers pertaining to different cellular pathways. Correlations between immunohistochemical markers and clinical factors, and among immunohistochemical markers, were verified. The discussions section aims to illustrate the potential biological implications of the immunophenotypic patterns we observed.

**Figure 5:**
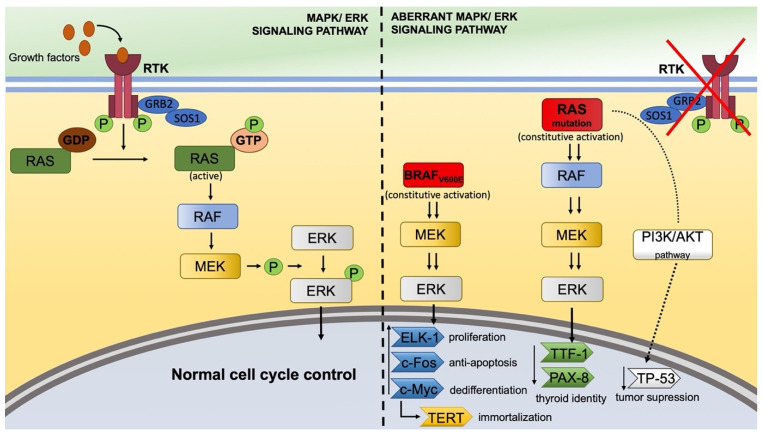
Normal (**left**) and aberrant (**right**) signaling pathways in thyroid carcinoma. The normal MAPK/ERK signaling pathway starts with growth factors that activate the RTK. Upon activation, its two intracellular domains cross-phosphorylate each other at the tyrosine residues. These phosphorylated residues activate GRB2, which recruits SOS1 adapter protein. SOS1 activates RAS, which begins the cascade by activating RAF, then MEK, which phosphorylates ERK. Phosphorylated ERK translocates inside the nucleus and controls the normal cell cycle. In the aberrant signaling pathway controlling thyroid carcinomas, the activation of the MAPK/ERK pathway works without the RTK and growth factors. BRAF_V600E_ mutant protein is constitutively activated and further supra-activates MEK and ERK. RAS mutations are also constitutively activated and subsequently activate RAF, MEK, and ERK. This leads to the increase of different nuclear transcription factors such as ELK-1, c-Fos, c-Myc, and a decrease of TTF-1 and PAX-8, which results in thyroid identity loss and RAI refractoriness. c-Myc can activate the transcription of TERT, or a TERT promoter mutation leads to cell immortalization. Mutant RAS can also decrease TP-53 via PI3K-AKT pathway, resulting in loss of tumor suppression and chaotic cell cycle.

We based our panel of markers by following protein components with phenotypic expression pertaining to the MAPK/ERK pathway and PI3K/AKT. The normal MAPK/ERK signaling pathway can be seen in Figure 5. Normally, the thyroid follicular cell relies on growth factors such as thyroid-stimulating hormone, insulin-like growth factor, and epidermal growth factor to activate the MAPK/ERK signaling pathway [31,43,44]. The binding of such a growth factor to its receptor promotes its dimerization and activation of the intracellular kinase domain [45]. Through the adaptor proteins, RAS gets activated, followed by RAF and MEK [46,47]. MEK phosphorylates ERK, which is translocated inside the nucleus where it exerts its effect on numerous transcription factors [48]. While we discuss downstream effects such as proliferation, dedifferentiation, and invasiveness, it should be emphasized that these are inferred from established literature and known roles of these pathways. Our study did not directly measure these functional outcomes.

In the neoplastic cell, this signaling cascade can be disrupted at different levels by mutant proteins through different mechanisms of action [46]. BRAF mutation, specifically V600E mutation, activates the cascade in a receptor-independent manner, resulting in vigorous aberrant activation and loss of normal cycle control, paired with anarchic proliferation via the nuclear transcription factors [46].

In our case, ATC was significantly associated with a higher prevalence of BRAF_V600E_ mutant protein expression, as shown by the immunohistochemical study. This finding is consistent with prior scientific reports from the literature describing higher mutational burdens for ATC, especially concerning BRAF_V600E_ mutation [22,49,50]. This is true for genetic testing for aberrant BRAF_V600E_. The immunohistochemical study of Haiyan et al. provides the same conclusion regarding prevalence but also supports the stepwise origin of cancer by demonstrating very close BRAF_V600E_ staining activities in the ATC or PDTC and the associated regions of differentiated thyroid carcinomas [51].

An interesting finding in our study was that total BRAF protein staining quantity is negatively correlated with specific BRAF_V600E_ staining levels. This contrasts with the literature—largely focusing on papillary carcinomas—where cases harboring BRAF_V600E_ mutation typically show higher total BRAF levels [19]. In line with this, Bertol and Chai also analyzed BRAF mRNA levels and similarly observed an elevation in BRAF in BRAF_V600E_-mutant tumors [19,52].

Several factors, however, could lead to our contrasting conclusion, one of them being the possibility of a technical artifact [53]. Antibody specificity could be different: the specific BRAF_V600E_ antibody only recognizes the mutant form (glutamic acid instead of valine at codon 600), whereas the total BRAF can bind to wild-type or mutant protein. Discordance may result if the antibodies differ in sensitivity, epitope affinity, or cross-reactivity. Also, uneven fixation times between tissue samples and antigen retrieval steps may alter the antigenicity and subsequent tissue staining [53,54]. Fixation times may induce variability among literature reports as delayed fixation and underfixation (<12 h) can greatly impair VE1 clone activity [55]. In our case, fixation was considered optimal as immersion in buffered formalin occurred in a few minutes and was done for 24 h. Manual processing versus automated staining, along with different chromogenic substrates, may impart variability among studies. Staining interpretation systems also vary highly between different writings [55]. All these findings indicate that total BRAF expression may be of limited diagnostic value, and better results can be achieved by using BRAF_V600E_-specific antibodies, thus in a standardized manner and further research should address this topic [55,56].

An opposite conclusion is generated by Kondo et al., who prove that, in their lot of 15 patients with ATC, the total BRAF expression is independent of BRAF_V600E_ mutation status and it has a heterogeneous expression, with nearly 50% being negative for immunophenotypic expression [57]. The same conclusion is forwarded by Khan et al., who show on Western blot that the expression levels of BRAF are independent of the mutational status [58]. It is also mandatory to mention that other genomic modifications, such as rearrangements resulting in copy number gain, can be the driver mutation behind BRAF aberration [59]. All these findings should be interpreted cautiously because none of them is dedicated to aggressive thyroid carcinomas like ATC and PDTC, the majority being focused on papillary thyroid carcinomas.

BRAF mutations, specifically BRAF_V600E,_ were highly researched, and a link between radioiodine refractoriness and this alteration has been established. BRAF_V600E_ mutations silence NIS gene transcription, impairing normal localization of the symporter into the membrane [60,61]. It seems that this effect is carried on through a mechanism involving histone deacetylase upregulation, subsequent acetylation of the NIS promoter, leading inherently to its underexpression [12]. All these aspects converge on tumor dedifferentiation to PDTC or ATC, by progressive loss of thyroid differentiation markers. Although studies show that BRAF_V600E_ mutation reduces radioiodine response, there is not enough evidence to recommend molecular or immunohistochemical postoperative testing, as per American Thyroid Association Management Guidelines [62]. Consensus Statement Recommendations on Actionable Biomarker Testing for Thyroid Cancer Management currently recommends fast specific anti-BRAF_V600E_, as inhibitors may exert better clinical outcomes if administered early [63].

Another aberrant switch-on in the pathway is the RAS mutant protein, which is constitutively activated and resistant to GTPase [46]. This mutation is associated with poor prognosis, aggressive behavior, and dedifferentiation through chromosomal instability [64,65,66]. In our lot, RAS expression was correlated with total BRAF expression, which may reflect the downstream nuclear effects. Total BRAF also correlates with TERT nuclear expression, as expected, because BRAF results in an increase of c-Myc transcription factor, which directly activates TERT [67,68]. We only tested the K-RAS isoform of RAS because this mutation may delimitate a distinct clinicopathologic entity [69]. Furthermore, studies are isolating candidate molecules for K-RAS cascade inhibition usable for future treatments [70,71]. This can make K-RAS an actionable target, mainly in the case of aggressive thyroid carcinomas, where therapeutic possibilities are limited.

TTF-1 and PAX-8 are both transcription factors that encode thyroid identity (e.g., thyroglobulin transcription, sodium/iodine symporter transcription, etc.) that tend to be lost in aggressive thyroid carcinomas [72]. In our study, we found a strong positive correlation between TTF-1 and PAX-8 nuclear expression, so we expect them to be somehow linked. Our immunohistochemical conclusion is in agreement with Dupain et al., who demonstrate on cell cultures that TTF-1 and PAX-8 reciprocally induce each other’s expression [36]. They also demonstrate that TTF-1 may have a more prominent role than PAX-8, being also more sensitive to epigenetic events, highlighting its importance as a predictive factor for aggressive forms of thyroid malignancy.

We observed that *BRAF*_V600E_ mutation significantly associates with *TERT* (C228T), both on the molecular analysis and immunohistochemistry, thus cooperatively contributing to the pathogenesis. There are many descriptions of this co-occurrence in the literature, but exclusively on papillary thyroid carcinomas [73,74]. Meta-analysis studies show that among the studied co-mutations, the association between the aforementioned modifications yields the worst prognosis factors and the highest aggressiveness: disease progression, tumor recurrence, overall mortality, and higher metastasis rate, extrathyroidal extension and lymph node invasion.

BRAF_V600E_ mutation upregulates TERT and accelerates dedifferentiation and progression via MAPK → Fos/Myc pathway [75,76,77]. KRAS expression was negatively associated with neural-invasive behavior, and also vascular invasion correlated with microscopic extrathyroidal extension, conclusion in accordance with the study of Reilly et al. [18]. The observed immunophenotypic patterns suggest potential mechanistic consequences on cell cycle regulation and tumor aggressiveness; however, these interpretations are based on the literature and pathway knowledge rather than direct experimental measurement in our cohort.

Ki-67 was an independent predictor factor for aggressive histologic subtype alone. Various studies in the literature prove the utility of Ki-67 as a reliable marker for predicting invasive behavior, recurrence, and poor overall survival, this being in accordance with our observation that Ki-67 positively correlates with the presence of metastasis [38,78,79]. From all the individual predicting factors, the multivariate analysis, however, established that only TTF-1 and the presence of necrosis remain significant prognostic factors for aggressive histologic subtype, with Ki-67 losing its significance, but still retaining its important role as a marker for aggressiveness. Also, Ki-67, along with lymph node invasion, were both individual predictor factors for the presence of metastases on univariate analysis.

We also aimed to investigate a BRAF_V600E_-specific antibody (clone VE1) and a polyclonal anti-TERT antibody as immunohistochemical predictors of the corresponding molecular events. VE1 BRAF_V600E_ antibody proves to be a sufficiently reliable tool for screening patients for actionable BRAF mutations via molecular techniques. Although we obtained poorer test efficacy parameters, we align with actual literature and recommend VE1 as a possible screening tool for BRAF_V600E_ mutation [56,80,81]. In the case of TERT, immunohistochemistry did not manage to achieve acceptable test parameters.

This study is limited by the retrospective design and relatively small cohort size, which may result in loss of statistical power and potential generalizability. Furthermore, the entire possibilities of correlations between clinical characteristics and immunohistochemical profiling was reduced by the lack of survival data. The immunohistochemical scoring system may be prone to interobserver variability. Although correlations were established, the causal relationship between marker expression and tumor progression remains to be proven. Further studies are needed to investigate the longitudinal molecular aspects and to assess and potentially confirm the causality between the molecular cascade components and validate panels in terms of diagnostic performance.

## 5. Conclusions

In conclusion, aggressive thyroid carcinomas in this cohort showed an immunophenotypic profile characterized by frequent BRAF_V600E_ mutant protein presence, total BRAF or K-RAS expression, frequent TERT overexpression, PAX-8 retention paired with TTF-1 loss, mutant TP53 pattern, and high Ki-67 indexes. Molecular profile confirms frequent BRAFV600E mutation and relatively low TERT C228T/C250T alterations. Specific anti-BRAFV600E mutant protein immunohistochemistry shows acceptable reliability for screening when compared to the molecular analysis. We identify an association between BRAFV600E and TERTC228 mutations, mainly in the case PDTC, thus identifying a possible mutational signature.

On univariate analysis, Ki-67, loss of TTF-1 expression, and presence of necrosis appeared to be reliable markers for anaplastic transformation. However, on multivariate analysis, only loss of TTF-1 and presence of necrosis remained independent predictors for anaplastic dedifferentiation, while Ki-67 did not retain statistical significance after adjustment. Ki-67 and lymph node invasion were independent predictor factors for presence of metastasis, but on multivariate analysis only lymph node invasion retained its significance.

Correlations between BRAF/RAS, TERT, and TP53 suggest entangled molecular dysregulation rather than a singular pathway. Incorporating a comprehensive immunohistochemical panel including BRAF_V600E_, total BRAF, TERT, PAX-8, TTF-1, TP53, and Ki-67 may help enhance diagnostic accuracy and could assist in prognostic stratification of aggressive thyroid carcinomas, though further validation is needed.

## Figures and Tables

**Table 1:** Antibody characteristics and dilutions.

	Antibody	Clone	Dilution	Producer	Ref. Number
1.	BRAF_v600E_	VE 1	1:100	Abcam, Cambridge, UK	ab228461
2.	B-RAF	EP152Y	1:70	Abcam, Cambridge, UK	ab33899
3.	K-RAS	EPR23474-20	1:70	Abcam, Cambridge, UK	ab275875
4.	TERT	Poly	1:70	Abcam, Cambridge, UK	ab216625
5.	PAX-8	PAX8R1	1:200	Abcam, Cambridge, UK	ab53490
6.	TTF1	SPT24	RTU	Leica Biosystems, Deer Park, IL, USA	PA0364
7.	P53	DO-7	RTU	Leica Biosystems, Deer Park, IL, USA	PA0057
8.	Ki-67	MM1	RTU	Leica Biosystems, Deer Park, IL, USA	PA0118

**Table 2:** Cut-off values for markers and their interpretation.

Marker	Cut off	Interpretation	Result
**BRAF_v600E_** **BRAF** **K-RAS** **TERT** **PAX-8**	No positive cells	+0	Qualitative
	Rare/isolated cells	+1	
	Focal/zonal positive	+2	
	Diffuse positive	+3	
**TTF1**	0%	Negative	Semiquantitative
	0–15%	Low	
	15-50%	Intermediate	
	>50%	High	
**Ki-67**	<5%	Low	
	5–10%	Intermediate	
	10–20%	High	
	>20%	Very high	
**P53**	0% or >10%	Mutant-type pattern	
	>0% and <10%	Wild-type pattern	

**Table 3:** Clinicopathological characteristics of the included patients and *p*-value.

Type of carcinoma		Total N = 40 (%)	PDTC N = 28 (%)	ATC N = 12 (%)	*p* Value
**Sex**	M	13 (32.5)	8 (28.8)	5 (58.3)	0.418
	F	27 (67.5)	20 (71.4)	7 (41.7)	
**Mean age (years)**		63.9 (39–83)	62.9 (39–83)	66.4 (46–83)	0.383
**Coexistence with DTC**		5 (12.5)	5 (17.8)	0 (0)	
**Necrosis present**		12 (30)	5 (17.8)	7 (58.3)	0.047 *
**Vascular invasion**		35 (87.5)	25 (89.2)	10 (83.3)	0.884
**Neural invasion**		12 (30)	8 (28.5)	4 (33.3)	0.646
**Extrathyroidal disease**		30 (81.1%)	22 (78.6%)	8 (88.9%) ^1^	0.492
**AJCC** **stage**	I		4 (14.3)	0	0.001 *
	II		13 (46.4)	0	
	III		2 (7.1)	0	
	IV IVA, IVB, IVC		8 (28.5) 1, 6, 0 ^2^	12 (100) 0 (0.00), 5 (71.4), 2 (28.6) ^3^	
**Guo et al., 2025** **[15]** **stage**	I		3 (10.7)		
	II		7 (25.0)		
	III		7 (25.0)		
	IV		11 (39.3)		
	IVA, IVB		6, 5		

^1^ three patients were excluded due to lack of tumoral stage; ^2^ one patient had an ambiguous subclass of stage IV and another patient could not be staged due to missing data; ^3^ five patients were excluded from the analysis due to the ambiguous subclass of stage IV, * statistically significant.

**Table 4:** Spearman correlation coefficients and interpretation between immunohistochemical and clinicopathologic data.

Variable 1	Variable 2	Spearman’s ρ	*p*-Value
**Immunohistochemical marker—immunohistochemical marker correlations**			
BRAF_V600E_	BRAF	−0.324	<0.05
BRAF	K-RAS	0.711	<0.001
BRAF	TERT	0.422	<0.01
TERT	PAX-8	0.323	<0.05
TTF-1	PAX-8	0.711	<0.001
Ki-67	TERT	0.346	<0.05
Ki-67	BRAF	0.350	<0.05
**Clinical—immunohistochemical marker correlations**			
Diagnostic	Ki-67	0.383	<0.05
Diagnostic	TTF-1	−0.542	<0.001
Diagnostic	Necrosis	0.405	<0.01
Ki-67	Metastases	0.432	<0.05
BRAF_V600E_	Age	0.325	<0.05
K-RAS	Neural invasion	−0.368	<0.05
Vascular invasion	Microscopic ETE	0.480	<0.01
Lymph node	Metastasis	0.465	<0.01
**Clinical—molecular or immunohistochemical—molecular correlations**			
BRAF_V600E_ histo	*BRAF*_V600E_ molecular	0.420	<0.01
BRAF_V600E_ histo	*TERT* C228T molecular	0.328	<0.05
*BRAF*_V600E_ molecular	*TERT* C228T molecular	0.329	<0.05

## Data Availability

Data presented in this study are available upon reasonable request from the corresponding author.

## References

[1] Ferlay J., Ervik M., Lam F., Laversanne M., Colombet M., Mery L., Piñeros M., Znaor A., Soerjomataram I., Bray F. (2024). Global Cancer Observatory: Cancer Today.

[2] Gao M.Z., Omer T.M., Miller K.M., Simpson M.C., Bukatko A.R., Gedion K., Boakye E.A., Kost K.M., Dickinson J.A., Varvares M.A. (2025). Thyroid Cancer Incidence and Trends in United States and Canadian Pediatric, Adolescent, and Young Adults. Cancers.

[3] Lebbink C.A., Links T.P., Czarniecka A., Dias R.P., Elisei R., Izatt L., Krude H., Lorenz K., Luster M., Newbold K. (2022). 2022 European Thyroid Association Guidelines for the management of pediatric thyroid nodules and differentiated thyroid carcinoma. Eur. Thyroid. J..

[4] Bernier M.-O., Withrow D.R., de Gonzalez A.B., Lam C.J.K., Linet M.S., Kitahara C.M., Shiels M.S. (2019). Trends in pediatric thyroid cancer incidence in the United States, 1998–2013. Cancer.

[5] Kitahara C.M., Schneider A.B. (2022). Epidemiology of Thyroid Cancer. Cancer Epidemiol. Biomark. Prev..

[6] Pellegriti G., Frasca F., Regalbuto C., Squatrito S., Vigneri R. (2013). Worldwide Increasing Incidence of Thyroid Cancer: Update on Epidemiology and Risk Factors. J. Cancer Epidemiol..

[7] Rodrigues R.F., Roque L., Krug T., Leite V. (2007). Poorly differentiated and anaplastic thyroid carcinomas: Chromosomal and oligo-array profile of five new cell lines. Br. J. Cancer.

[8] Molinaro E., Romei C., Biagini A., Sabini E., Agate L., Mazzeo S., Materazzi G., Sellari-Franceschini S., Ribechini A., Torregrossa L. (2017). Anaplastic thyroid carcinoma: From clinicopathology to genetics and advanced therapies. Nat. Rev. Endocrinol..

[9] Bhattacharya S., Mahato R.K., Singh S., Bhatti G.K., Mastana S.S., Bhatti J.S. (2023). Advances and challenges in thyroid cancer: The interplay of genetic modulators, targeted therapies, and AI-driven approaches. Life Sci..

[10] WHO Classification of Tumours Editorial Board (2022). Endocrine and Neuroendocrine Tumours.

[11] Schipor S., Publik M.A., Manda D., Ceausu M. (2025). Aggressive Thyroid Carcinomas Clinical and Molecular Features: A Systematic Review. Int. J. Mol. Sci..

[12] Hsu K.-T., Yu X.-M., Audhya A.W., Jaume J.C., Lloyd R.V., Miyamoto S., Prolla T.A., Chen H. (2014). Novel Approaches in Anaplastic Thyroid Cancer Therapy. Oncologist.

[13] Al-Ibraheem A., Al-Rasheed U., Mashhadani N., Abdlkadir A.S., Al-Adhami D.A., Ruzzeh S., Istatieh F., Mansour A., Hamdan B., Kheetan R. (2023). Long-Term Survival Analysis and Prognostic Factors of Arabic Patients with Differentiated Thyroid Carcinoma: A 20-Year Observational Study at the King Hussein Cancer Center (KHCC) Involving 528 Patients. Cancers.

[14] Patel J., Klopper J., Cottrill E.E. (2023). Molecular diagnostics in the evaluation of thyroid nodules: Current use and prospective opportunities. Front. Endocrinol..

[15] Guo Y., Zhong Q., Xie L., Wang X., Guo L., Zhuang S., Jia C., Wu L., Peng J., Pang F. (2025). Development of a new TNM staging system for poorly differentiated thyroid carcinoma: A multicenter cohort study. Front. Endocrinol..

[16] Cracolici V. (2023). No Longer Well-Differentiated: Diagnostic Criteria and Clinical Importance of Poorly Differentiated/High-Grade Thyroid Carcinoma. Surg. Pathol. Clin..

[17] BlueBooksOnline. https://tumourclassification.iarc.who.int/chaptercontent/53/47.

[18] Reilly J., Faridmoayer E., Lapkus M., Pastewski J., Sun F., Elassar H., Studzinski D.M., Callahan R.E., Czako P., Nagar S. (2022). Vascular invasion predicts advanced tumor characteristics in papillary thyroid carcinoma. Am. J. Surg..

[19] Bertol B.C., Massaro J.D., Debortoli G., Santos A.L.P., de Araújo J.N.G., Giorgenon T.M.V., e Silva M.C., de Figueiredo-Feitosa N.L., Collares C.V.A., de Freitas L.C.C. (2023). BRAF, TERT and HLA-G Status in the Papillary Thyroid Carcinoma: A Clinicopathological Association Study. Int. J. Mol. Sci..

[20] de Biase D., Torricelli F., Ragazzi M., Donati B., Kuhn E., Visani M., Acquaviva G., Pession A., Tallini G., Piana S. (2018). Not the same thing: Metastatic PTCs have a different background than ATCs. Endocr. Connect..

[21] Asioli S., A Erickson L., Righi A., Jin L., Volante M., Jenkins S., Papotti M., Bussolati G., Lloyd R.V. (2010). Poorly differentiated carcinoma of the thyroid: Validation of the Turin proposal and analysis of IMP3 expression. Mod. Pathol..

[22] Landa I., Ibrahimpasic T., Boucai L., Sinha R., Knauf J.A., Shah R.H., Dogan S., Ricarte-Filho J.C., Krishnamoorthy G.P., Xu B. (2016). Genomic and transcriptomic hallmarks of poorly differentiated and anaplastic thyroid cancers. J. Clin. Investig..

[23] Xu B., Fuchs T.L., Dogan S., Landa I., Katabi N., Fagin J.A., Tuttle R.M., Sherman E.J., Gill A.J., Ghossein R. (2020). Dissecting Anaplastic Thyroid Carcinoma: A Comprehensive Clinical, Histologic, Immunophenotypic, and Molecular Study of 360 Cases. Thyroid.

[24] Lam K.Y., Lo C.Y., Chan K.W., Wan K.Y. (2000). Insular and anaplastic carcinoma of the thyroid: A 45-year comparative study at a single institution and a review of the significance of p53 and p21. Ann. Surg..

[25] Xu B., Ghossein R. (2016). Genomic Landscape of poorly Differentiated and Anaplastic Thyroid Carcinoma. Endocr. Pathol..

[26] Kondo T., Ezzat S., Asa S.L. (2006). Pathogenetic mechanisms in thyroid follicular-cell neoplasia. Nat. Rev. Cancer.

[27] De Leo S., Trevisan M., Fugazzola L. (2020). Recent advances in the management of anaplastic thyroid cancer. Thyroid. Res..

[28] Masui T., Yane K., Ota I., Kakudo K., Wakasa T., Koike S., Kinugawa H., Yasumatsu R., Kitahara T. (2025). Low Ki-67 labeling index is a clinically useful predictive factor for recurrence-free survival in patients with papillary thyroid carcinoma. J. Pathol. Transl. Med..

[29] Li L.T., Jiang G., Chen Q., Zheng J.N. (2014). Ki67 is a promising molecular target in the diagnosis of cancer (Review). Mol. Med. Rep..

[30] Seethala R.R., Asa S.L., Bullock M.J., Carty S.E., Hodak S.P., McHugh J.B., Nikiforov Y.E., Pettus J., Richardson M.S., Shah J. (2017). Protocol for the Examination of Specimens from Patients with Carcinomas of the Thyroid Gland.

[31] Talbott I., Wakely P.E. (2015). Undifferentiated (anaplastic) thyroid carcinoma: Practical immunohistochemistry and cytologic look-alikes. Semin. Diagn. Pathol..

[32] Crescenzi A., Baloch Z. (2023). Immunohistochemistry in the pathologic diagnosis and management of thyroid neoplasms. Front. Endocrinol..

[33] Dettmer M.S., Schmitt A., Komminoth P., Perren A. (2020). Gering differenzierte Schilddrüsenkarzinome: Eine unterdiagnostizierte Entität. Der Pathol..

[34] Walczyk A., Kopczyński J., Gąsior-Perczak D., Pałyga I., Kowalik A., Chrapek M., Hejnold M., Góźdź S., Kowalska A. (2020). Histopathology and immunohistochemistry as prognostic factors for poorly differentiated thyroid cancer in a series of Polish patients. PLoS ONE.

[35] Bishop J.A., Sharma R., Westra W.H. (2011). PAX8 immunostaining of anaplastic thyroid carcinoma: A reliable means of discerning thyroid origin for undifferentiated tumors of the head and neck. Hum. Pathol..

[36] Induction of TTF-1 or PAX-8 Expression on Proliferation and Tumorigenicity in Thyroid Carcinomas. https://www.spandidos-publications.com/ijo/49/3/1248.

[37] Lacka K., Maciejewski A., Tyburski P., Manuszewska-Jopek E., Majewski P., Więckowska B. (2025). Rationale for Testing TP53 Mutations in Thyroid Cancer—Original Data and Meta-Analysis. Int. J. Mol. Sci..

[38] Harahap W.A., Tofrizal T., Oktahermoniza O. (2022). Relationship between the Expression of BRAF V600E and Ki-67 with the Recurrence of Well-Differentiated Thyroid Cancer. Asian Pac. J. Cancer Prev..

[39] Volante M., Collini P., Nikiforov Y.E., Sakamoto A., Kakudo K., Katoh R., Lloyd R.V., LiVolsi V.A., Papotti M., Sobrinho-Simoes M. (2007). Poorly differentiated thyroid carcinoma: The Turin proposal for the use of uniform diagnostic criteria and an algorithmic diagnostic approach. Am. J. Surg. Pathol..

[40] Szymonek M., Kowalik A., Kopczyński J., Gąsior-Perczak D., Pałyga I., Walczyk A., Gadawska-Juszczyk K., Płusa A., Mężyk R., Chrapek M. (2017). Immunohistochemistry cannot replace DNA analysis for evaluation of *BRAF*V600E mutations in papillary thyroid carcinoma. Oncotarget.

[41] Bedekovics J., Madarász K., Mokánszki A., Molnár S., Mester Á., Miltényi Z., Méhes G. (2024). Exploring p53 protein expression and its link to *TP53* mutation in myelodysplasia-related malignancies—Interpretive challenges and potential field of applications. Histopathology.

[42] Hwang H.J., Nam S.K., Park H., Park Y., Koh J., Na H.Y., Kwak Y., Kim W.H., Lee H.S. (2020). Prediction of TP53 mutations by p53 immunohistochemistry and their prognostic significance in gastric cancer. J. Pathol. Transl. Med..

[43] Ouh Q.-Y., Grossman R.F. (1995). Thyroid Growth Factors, Signal Transduction Pathways, and Oncogenes. Surg. Clin. N. Am..

[44] Smith T.J. (2021). Insulin-Like Growth Factor Pathway and the Thyroid. Front. Endocrinol..

[45] ERK/MAPK Signalling Pathway and Tumorigenesis (Review). https://www.spandidos-publications.com/10.3892/etm.2020.8454.

[46] Burotto M., Chiou V.L., Lee J., Kohn E.C. (2014). The MAPK pathway across different malignancies: A new perspective. Cancer.

[47] Sun Y., Liu W.-Z., Liu T., Feng X., Yang N., Zhou H.-F. (2015). Signaling pathway of MAPK/ERK in cell proliferation, differentiation, migration, senescence and apoptosis. J. Recept. Signal Transduct..

[48] Maik-Rachline G., Hacohen-Lev-Ran A., Seger R. (2019). Nuclear ERK: Mechanism of Translocation, Substrates, and Role in Cancer. Int. J. Mol. Sci..

[49] Duan H., Li Y., Hu P., Gao J., Ying J., Xu W., Zhao D., Wang Z., Ye J., Lizaso A. (2019). Mutational profiling of poorly differentiated and anaplastic thyroid carcinoma by the use of targeted next-generation sequencing. Histopathology.

[50] Yamazaki H., Kunisaki C., Sugimori M., Rino Y., Saito A. (2024). Genetic landscape of 482 thyroid carcinomas: Analysis with the national datacenter for cancer genomic medicine in Japan. Endocrine.

[51] Gu H., Wang J., Ran W., Li G., Hu S., Zhao H., Wang X., Wang J. (2024). Anaplastic and poorly differentiated thyroid carcinomas: Genetic evidence of high-grade transformation from differentiated thyroid carcinoma. J. Pathol. Clin. Res..

[52] Chai Y.J., Yi J.W., Jee H.-G., A Kim Y., Kim J.H., Xing M., Lee K.E. (2016). Significance of the BRAF mRNA Expression Level in Papillary Thyroid Carcinoma: An Analysis of The Cancer Genome Atlas Data. PLoS ONE.

[53] Khan S., Bhake A., Sagar S., Yelne S. (2024). Deciphering the Role of BRAFV600E Immunohistochemistry in Breast Lesions: A Comprehensive Review. Cureus.

[54] Tsutsumi Y. (2021). Pitfalls and Caveats in Applying Chromogenic Immunostaining to Histopathological Diagnosis. Cells.

[55] Parker K.G., White M.G., Cipriani N.A. (2020). Comparison of Molecular Methods and BRAF Immunohistochemistry (VE1 Clone) for the Detection of BRAF V600E Mutation in Papillary Thyroid Carcinoma: A Meta-Analysis. Head Neck Pathol..

[56] Dvorak K., Aggeler B., Palting J., McKelvie P., Ruszkiewicz A., Waring P. (2014). Immunohistochemistry with the anti-BRAF V600E (VE1) antibody: Impact of pre-analytical conditions and concordance with DNA sequencing in colorectal and papillary thyroid carcinoma. Pathology.

[57] Kondo T., Nakazawa T., Murata S.-I., Kurebayashi J., Ezzat S., Asa S.L., Katoh R. (2007). Enhanced B-Raf protein expression is independent of V600E mutant status in thyroid carcinomas. Hum. Pathol..

[58] Khan M.S., Pandith A.A., Azad N., Hussain M.U., Masoodi S.R., Wani K.A., Andrabi K.I., Mudassar S. (2014). Impact of molecular alterations of BRAF in the pathogenesis of thyroid cancer. Mutagenesis.

[59] Ciampi R., Zhu Z., Nikiforov Y.E. (2005). BRAF copy number gains in thyroid tumors detected by fluorescence in situ hybridization. Endocr. Pathol..

[60] Riesco-Eizaguirre G., Gutiérrez-Martínez P., García-Cabezas M.A., Nistal M., Santisteban P. (2006). The oncogene BRAFV600E is associated with a high risk of recurrence and less differentiated papillary thyroid carcinoma due to the impairment of Na+/I− targeting to the membrane. Endocr.-Relat. Cancer.

[61] Romei C., Ciampi R., Faviana P., Agate L., Molinaro E., Bottici V., Basolo F., Miccoli P., Pacini F., Pinchera A. (2008). BRAFV600E mutation, but not RET/PTC rearrangements, is correlated with a lower expression of both thyroperoxidase and sodium iodide symporter genes in papillary thyroid cancer. Endocr.-Relat. Cancer.

[62] Haugen B.R., Alexander E.K., Bible K.C., Doherty G.M., Mandel S.J., Nikiforov Y.E., Pacini F., Randolph G.W., Sawka A.M., Schlumberger M. (2016). 2015 American Thyroid Association Management Guidelines for Adult Patients with Thyroid Nodules and Differentiated Thyroid Cancer: The American Thyroid Association Guidelines Task Force on Thyroid Nodules and Differentiated Thyroid Cancer. Thyroid.

[63] Mete O., Boucher A., Schrader K.A., Abdel-Rahman O., Bahig H., Ho C., Hasan O.K., Lemieux B., Winquist E., Wong R. (2024). Consensus Statement: Recommendations on Actionable Biomarker Testing for Thyroid Cancer Management. Endocr. Pathol..

[64] I Saavedra H., A Knauf J., Shirokawa J.M., Wang J., Ouyang B., Elisei R., Stambrook P.J., A Fagin J. (2000). The RAS oncogene induces genomic instability in thyroid PCCL3 cells via the MAPK pathway. Oncogene.

[65] Garcia-Rostan G., Zhao H., Camp R.L., Pollan M., Herrero A., Pardo J., Wu R., Carcangiu M.L., Costa J., Tallini G. (2003). *ras* Mutations Are Associated With Aggressive Tumor Phenotypes and Poor Prognosis in Thyroid Cancer. J. Clin. Oncol..

[66] Feng L., Li M., Zhang Q.-P., Piao Z.-A., Wang Z.-H., Lv S. (2011). Utility of BRAF protein overexpression in predicting the metastasis potential of papillary thyroid carcinoma. Oncol. Lett..

[67] Yuan X., Larsson C., Xu D. (2019). Mechanisms underlying the activation of TERT transcription and telomerase activity in human cancer: Old actors and new players. Oncogene.

[68] Tsai W.-B., Aiba I., Long Y., Lin H.-K., Feun L., Savaraj N., Kuo M.T. (2012). Activation of Ras/PI3K/ERK Pathway Induces c-Myc Stabilization to Upregulate Argininosuccinate Synthetase, Leading to Arginine Deiminase Resistance in Melanoma Cells. Cancer Res..

[69] Radkay L.A., Chiosea S.I., Seethala R.R., Hodak S.P., LeBeau S.O., Yip L., McCoy K.L., Carty S.E., Schoedel K.E., Nikiforova M.N. (2014). Thyroid nodules with *KRAS* mutations are different from nodules with *NRAS* and *HRAS* mutations with regard to cytopathologic and histopathologic outcome characteristics. Cancer Cytopathol..

[70] Tayubi I.A., Madar I.H. (2023). Identification of potential inhibitor targeting KRAS mutation in Papillary Thyroid Carcinoma through molecular docking and dynamic simulation analysis. Comput. Biol. Med..

[71] Mannino D., Basilotta R., De Luca F., Casili G., Esposito E., Paterniti I. (2025). KRAS–SOS-1 Inhibition as New Pharmacological Target to Counteract Anaplastic Thyroid Carcinoma (ATC). Int. J. Mol. Sci..

[72] Di Magliano M.P., Di Lauro R., Zannini M. (2000). Pax8 has a key role in thyroid cell differentiation. Proc. Natl. Acad. Sci. USA.

[73] Nhung N.T., Hoang V.D., Mussazhanova Z., Kurohama H., Ha L.N., Matsuda K., Nguyen V.P.T., Hanh N.T.M., Nguyen T.N.A., Nakashima M. (2025). BRAFV600E and TERT promoter mutations and their impact on recurrent papillary thyroid carcinoma progression. Endocr. Connect..

[74] Rusinek D., Pfeifer A., Krajewska J., Oczko-Wojciechowska M., Handkiewicz-Junak D., Pawlaczek A., Zebracka-Gala J., Kowalska M., Cyplinska R., Zembala-Nozynska E. (2018). Coexistence of *TERT* Promoter Mutations and the *BRAF* V600E Alteration and Its Impact on Histopathological Features of Papillary Thyroid Carcinoma in a Selected Series of Polish Patients. Int. J. Mol. Sci..

[75] Yu P., Qu N., Zhu R., Hu J., Han P., Wu J., Tan L., Gan H., He C., Fang C. (2023). TERT accelerates BRAF mutant–induced thyroid cancer dedifferentiation and progression by regulating ribosome biogenesis. Sci. Adv..

[76] Liu R., Zhang T., Zhu G., Xing M. (2018). Regulation of mutant TERT by BRAF V600E/MAP kinase pathway through FOS/GABP in human cancer. Nat. Commun..

[77] Chung J.H. (2020). BRAF and TERT promoter mutations: Clinical application in thyroid cancer. Endocr. J..

[78] Pan D.-H., Wen D.-Y., Luo Y.-H., Chen G., Yang H., Chen J.-Q., He Y. (2017). The diagnostic and prognostic values of Ki-67/MIB-1 expression in thyroid cancer: A meta-analysis with 6,051 cases. OncoTargets Ther..

[79] Chowdhury R., Alsayegh R., Forest V.-I., Pusztaszeri M.P., da Silva S.D., Florianova L., Payne R.J. (2024). Ki-67 Labelling Index as a Predictor of Invasive Features in Thyroid Cancer: Retrospective Analysis and Implications. Curr. Oncol..

[80] Lee S.-R., Yim H., Han J.H., Lee K.B., Lee J., Soh E.Y., Kim D.J., Chung Y.-S., Jeong S.-Y., Sheen S.S. (2015). VE1 Antibody Is Not Highly Specific for the *BRAF* V600E Mutation in Thyroid Cytology Categories With the Exception of Malignant Cases. Am. J. Clin. Pathol..

[81] Schafroth C., Galván J.A., Centeno I., Koelzer V.H., Dawson H.E., Sokol L., Rieger G., Berger M.D., Hädrich M., Rosenberg R. (2015). VE1 immunohistochemistry predicts *BRAF*V600E mutation status and clinical outcome in colorectal cancer. Oncotarget.

